# 703. Peritoneal Coccidioidomycosis in a Pediatric Patient: An Extremely Rare Presentation and Literature Review

**DOI:** 10.1093/ofid/ofab466.900

**Published:** 2021-12-04

**Authors:** Barbara Ximenes Braz, Chantal Soobhanath, Amelia B Thompson

**Affiliations:** 1 Adventhealth Orlando, Orlando, Florida; 2 AdventHealth for Children, Orlando, Florida

## Abstract

**Background:**

Chronic peritonitis is an unusual manifestation of coccidiomycosis (CM) that is challenging to diagnose and manage due to its propensity for relapse. It is even more unusual to diagnose peritoneal CM in the pediatric population, with only two other cases reported in the literature.

**Methods:**

We present the case of a previously healthy 5-year-old Filipino female in Florida who was diagnosed with peritoneal CM. After months of unintentional weight loss and worsening abdominal distention, she presented to medical care. Imaging revealed significant abdominal ascites and nodularities throughout the peritoneum. The peritoneal fluid demonstrated a lymphocytic pleocytosis and infectious workup was benign. CA125 levels were elevated, but peritoneal adenosine deaminase was within normal limits. A biopsy of the affected tissue revealed diffuse granulomas surrounding spherules that were positive on GMS and PAS staining, concerning for CM. Exposure history revealed that she was raised in California and moved to Florida one year prior to presentation. Complement fixation titers were significantly elevated at ≥ 1:512 and immunodiffusion titers were positive. A Coccidioides PCR was sent from the tissue to the Mayo clinic and was positive, and fungal cultures from the tissue grew *C. immitis/posadasii.* Immunologic workup was reassuring. She was started on oral Fluconazole with rapid resolution of her symptoms.

**Results:**

Involvement of the peritoneum in CM is extremely rare. Abdominal distention due to ascites is the most common presentation, and the peritoneal fluid is typically exudative. Imaging may reveal peritoneal deposits which can mimic other infections and malignancy. Diagnosis can be based on histopathological demonstration of fungal structures, cultures, antibody testing, antigen detection and/or PCR. Treatment guidelines suggest azole therapy for nonmeningeal disseminated CM with at least 6–12 months of treatment for extrapulmonary coccidioidal soft tissue infection.

**Conclusion:**

Peritoneal CM is an extremely uncommon condition and it is even more rare in the pediatric population, but should be considered in those in the appropriate clinical settings, particularly if they have history to suggest exposure to regions where this fungus is endemic.

**Disclosures:**

**All Authors**: No reported disclosures

